# Effect of the *cagW*-based gene vaccine on the immunologic properties of BALB/c mouse: an efficient candidate for *Helicobacter pylori* DNA vaccine

**DOI:** 10.1186/s12951-020-00618-1

**Published:** 2020-04-21

**Authors:** Mohammad Chehelgerdi, Abbas Doosti

**Affiliations:** 1 Young Researchers and Elite Club, Shahrekord Branch, Islamic Azad University, Shahrekord, Iran; 2 Biotechnology Research Center, Shahrekord Branch, Islamic Azad University, Shahrekord, Iran

**Keywords:** BALB/c, Chitosan nanoparticles, DNA vaccine, *Helicobacter pylori*, Virulence factor

## Abstract

**Background:**

*Helicobacter pylori* (*H. pylori*) infect more than half of the world population, and they cause different serious diseases such as gastric carcinomas. This study aims to design a vaccine on the basis of *cagW* against *H. pylori* infection. After pcDNA3.1 (+)-*cagW*–CS-NPs complex is produced, it will be administered into the muscles of healthy BALB/c mice in order to study the effect of this DNA vaccine on the interleukin status of mice, representing its effect on the immune system. After that, the results will be compared with the control groups comprising the administration of *cagW*-pCDNA3.1 (+) vaccine, the administration of chitosan and the administration of PBS in the muscles of mice.

**Methods:**

The *cagW* gene of *H. pylori* was amplified by employing PCR, whose product was then cloned into the pcDNA3.1 (+) vector, and this cloning was confirmed by PCR and BamHI/EcoRV restriction enzyme digestion. *CagW* gene DNA vaccine was encapsulated in chitosan nanoparticles (pcDNA3.1 (+)-*cagW*-CS-NPs) using a complex coacervation method. The stability and in vitro expression of chitosan nanoparticles were studied by DNase I digestion and transfection, and the immune responses elicited in specific pathogen-free (SPF) mice by the pcDNA3.1 (+)-cagW-CS-NPs were evaluated. Apart from that, the protective potential pcDNA3.1 (+)-*cagW*-CS-NPs was evaluated by challenging with *H. pylori*.

**Results:**

The pcDNA3.1 (+)-*cagW*-CS-NPs comprises *cagW* gene of *H. pylori* that is encapsulated in chitosan nanoparticles, produced with good morphology, high stability, a mean diameter of 117.7 nm, and a zeta potential of + 5.64 mV. Moreover, it was confirmed that chitosan encapsulation protects the DNA plasmid from DNase I digestion, and the immunofluorescence assay showed that the *cagW* gene could express in HDF cells and maintain good bioactivity at the same time. In comparison to the mice immunized with the control plasmid, in vivo immunization revealed that mice immunized with pcDNA3.1 (+)-*cagW*-NPs showed better immune responses and prolonged release of the plasmid DNA.

**Conclusions:**

This research proves chitosan-DNA nanoparticles as potent immunization candidates against *H. pylori* infection and paves the way for further developments in novel vaccines encapsulated in chitosan nanoparticles.

## Background

Nanotechnology has become an interesting area in a wide variety of studies, and nanoparticles (NPs) have particularly attracted attention in medicine [1] due to their huge potential in specific drug delivery, combination therapy, low toxicity, low dosage of agents and minimal side effects. Poor vaccine delivery and its inactivation due to degradation by DNase lead to reduced immunogenicity by DNA vaccines. One of the advantageous applications of the NPs is related to vaccine delivery [2]. Hence, NPs can elevate the efficacy of DNA vaccine delivery and protect it from degradation in order to increase the DNA vaccine potency [3]. Actually, chitosan is able to combine with DNA vaccine in order to prepare a promising non-viral gene delivery system for DNA vaccine delivery [4]. It is a natural, biodegradable and biocompatible cationic copolymer that consists of β1-4 linked d-glucosamine and N-acetyl-d-glucosamine [5]. Furthermore, the capability of chitosan for gene delivery is associated with the positive charge, low toxicity and self-assembly with plasmid DNA (pDNA). Encapsulation of pDNA with chitosan polymer can protect pDNA from degradation in addition to controlling pDNA release [4]. Chitosan-pDNA complex has the potential to overcome issues surrounding transfection such as cell uptake, degradation in endo-lysosomes and plasmid trafficking into the nucleus [6]. The positive charge in chitosan compound also presents antimicrobial properties due to interaction with the negative charge on the bacterial cell surface. These interactions lead to reduced osmotic stability, membrane disruption and intracellular elements leakage [7]. However, it has also been reported that chitosan may enter the bacterial or fungal nuclei, thus preventing mRNA and protein synthesis by binding to their DNA [8].

Helicobacter pylori (*H. pylori*) is a Gram negative, spiral-shaped and micro-aerophilic bacterium infecting almost half of the world’s population. According to the World Health Organization reports, *H. pylori* is classified as a class I carcinogen [9]. Its colonization in the human gastric and duodenal mucosa probably gives rise to chronic gastritis, peptic ulcer and gastric mucosa-associated lymphoid tissue lymphoma (MALT) induction [10]. Currently, eradication of *H. pylori* infection is based on the combination of multiple antibiotics with a proton pump inhibitor which has shown effective improvements in 90% of cases [11]. However, antibiotic resistance crisis, along with the side effects of drug consumption, is the major concern about antibiotics prescription [12]. Therefore, DNA vaccination seems to be a promising strategy to deal with *H. pylori* infection. Recently, various candidate proteins have been identified as immunogenic agents in preclinical models such as urease B [13], heat shock proteins [14], vacuolating toxin A (vac A) [15], cytotoxin-associated antigen A (cag A) [16] and catalase [11]. Although these antigens are able to reduce the bacterial load in animal models, their protection against *H. pylori* infection is less than ideal. The cag Pathogenicity Island (cagPAI) is one of the major virulence factors in *H. pylori*-related diseases. The 40-kb cagPAI is a multi-operon region which encodes approximately 27 cag proteins. It has been reported that ~ 20 cag proteins are involved in type IV secretion system (T4SS) structure [17]. The T4SS is a versatile macro-molecular apparatus distributed in many bacterial species and introduced in multiple functions such as creating a conjugation system and translocation of DNA or other substrates (e.g., proteins and nucleo-protein complexes) to the host cells [18]. The well-known prototypical T4SS is the VirB/VirD4 system in *agrobacterium tumefaciens* that comprises a set of 11 VirB proteins (VirB1-VirB11) and a coupling protein VirD4. The VirB/VirD4 system is responsible for transferring the *A. tumefaciens* virulent proteins and T-DNA segment of tumor-inducing plasmid to the recipient plant cells [19]. In *H. pylori*, the T4SS is essential for transporting the bacterial onco-protein cagA into the epithelial cells, and it increases the malignant cell transformation and consequently the risk of gastric cancer [20]. Additionally, the T4SS transports peptidoglycan that induces the interleukin-8 secretion as a pro-inflammatory cytokine [17]. Some of the cag proteins share function, structure and/or sequence similarities with VirB/D4 system of *A. tumefaciens*. These VirB/D4 orthologs are necessary for cagA translocation through the T4SS. Although *cagW* (cag9/HP0529) do not share sequence similarities with VirB/D4 system components, some evidences obtained from protein–protein interaction, protein localization, and functional analysis suggest that *cagW* be the VirB6-analogue protein of the cag system and T4SS [21]. These evidences are mentioned as follows: (a) the VirB6 family members have 5–7 transmembrane helices, and the *cagW* also harbors 6 transmembrane helices; (b) the amino acid content of last predicted transmembrane helix in *cagW* is rich in valine/leucine/isoleucine, which is considered to be essential for VirB6 function; (c) both *cagW* and VirB6 encompass a substantial tryptophan residue within a conserved motif preceding the last predicted transmembrane helix 4, and (d) *cagW* structures multimer and its absence influences cellular levels of pilus forming components, *CagL*, *CagI* and *CagH*. Therefore, it has been proposed that the *cagW* fulfill an analogous function with VirB6 [21–23]. This study aims to enhance the efficacy of a DNA vaccine against *H. pylori* infections. A complex coacervation method was employed to construct *cagW* gene DNA vaccine encapsulated in chitosan nanoparticles (pcDNA3.1 (+)-*cagW*-CS-NPs). The stability and in vitro expression of the chitosan nanoparticles were studied by DNase I digestion and transfection, and the immune responses elicited in specific pathogen-free (SPF) mice by the pcDNA3.1 (+)-*cagW*-CS-NPs were evaluated. Furthermore, protective potential of pcDNA3.1 (+)-*cagW*-CS-NPs was assessed by challenging with *H.pylori*.

## Methods

### Ethical consideration

All animal protocols were performed in accordance with the Ethical Committee and Research Deputy of the Islamic Azad University of Shahrekord Branch, Iran for the Care and Use of Laboratory Animals, and were approved by the Institutional Animal Care and Use Committee guidelines of Islamic Azad University, Shahrekord, Iran (14th 2017 with ethics code: IR.IAU.SHK. REC.1397.045).

### Bacterial strains, cells and growth conditions

*Helicobacter pylori* strain (ATCC: 43504) was purchased from the Iranian Biological Resource Center (IBRC). This strain was cultivated on the Luria–Bertani (LB) agar)Sodium chloride, 5 g/l; yeast extract, 5 g/l; tryptone, 10 g/l; mixed with agar, 15 g/l) (Difco, USA) at 37 °C overnight. The HDF cells were provided by the National Cell Bank of Iran, Pasteur Institute and were grown in DMEM containing 10% fetal calf serum (FCS) (Gibco, US) with 5% CO_2_.

### DNA extraction and gene amplification

Bacterial DNA was isolated from *H. pylori* using a commercial DNA extraction kit (QIAamp^®^ DNA Mini Kit, Qiagen, USA) based on the manufacturer’s protocol. The quality of extracted DNA was analyzed by electrophoresis on a 1.0% agarose gel stained with ethidium bromide. DNA concentration was checked using the Thermo Scientific™ NanoDrop 2000 at a wavelength of 230, 260 and 280 nm. The specific primers for *cagW* gene (Accession Number: JQ685144.1) were designed by Beacon Designer version 7.91 to amplify a 1611 bp fragment. The primers had BamHI and EcoRV restriction sites in forward (TACGGATCCATGAAAAGGACTTTTTTAATAACG) and reverse primer (AACGATATCTTATCCTTTGAACATAGATCCAC), respectively. PCR amplification was carried out in a 25-μL reaction mixture of 1 µg template DNA, 2 mM MgCl2, 200μΜ dNTP mix, 2.5 µl of 10 × PCR buffer (20 mM Tris–HCl pH 8.4, 50 mM KCl), 1 µM of each primer and 1 unit of Taq DNA polymerase (Thermo Fisher Scientific, USA). For a negative control, 2 µl of sterile ultra-pure deionized water was used instead of template DNA. The thermal cycling was optimized with initial denaturation at 94 °C for 5 min followed by 33 cycles of denaturation at 95 °C for 1 min, annealing at 62 °C for 1 min, extension at 72 °C for 1 min, and ultimately a final extension at 72 °C for 10 min. Amplified PCR products were then analyzed by electrophoresis in 1.5% agarose gels.

### Construction of recombinant plasmids

The amplified *cagW* fragments were purified by Qiagen gel extraction kit (Qiagen, Germany) and ligated into plasmid vector pcDNA3.1 (+) using PCR cloning kit-Thermo Fisher Scientific according to manufacturer’s protocol. The cloning vector (pcDNA3.1 (+) plus *cagW*) was transformed into the competent cells *E. coli* TOP10F′ by calcium chloride (CaCl2) chemical method. To screen the recombinant vectors, the transformants were selected on LB-ampicillin agar plates. The presence of the *cagW* DNA insert was determined by screening bacterial colonies using PCR. The competent cells were plated on Luria–Bertani (LB) agar plates containing Xgal (20 mg/ml), IPTG (isopropyl-b-d-thiogalactoside) (0.1 M) and the plates were incubated at 30  °C overnight. The white colonies were picked and cultured again in LB broth media enriched with lg ml ampicillin at 30  °C for 10 h. The recombinant vector was purified using Plasmid Mini Extraction Kit (Bioneer, Korea) and was analyzed by specific- *cagW* primers. To confirm the accuracy of cloning, the recombinant vector was digested with BamHI and EcoRV restriction enzymes to confirm the presence of *cagW* fragment [24, 25].

### Preparation of chitosan solutions and plasmid DNA solutions

Chitosan (with a molecular weight of 71.3 kDa and deacetylation degree of 80%) was prepared from Sigma-Aldrich (Sigma, St Louis, MO, USA). To prepare 1.0% chitosan solutions, 1.0 g chitosan was dissolved into solution of 1.0% acetic acid. It is followed by adding acetate (5.0 mmol/l) to reach a final concentration of 250 μg/ml. A total concentration of 100 μg/ml pcDNA3.1 (+)-*cagW* plasmid DNA solutions was made by adding Na_2_SO_4_ solution (5.0 mmol/l).

### Preparation of the pcDNA3.1 (+)-*cagW*-CS-NPs

A complex coacervation method was applied to form the plasmid DNA chitosan nanoparticles. First, both chitosan solutions and the plasmid DNA solutions were located in a water bath of 55 °C for 30 min. Then, the mixture of plasmid DNA solution and the chitosan was blended for 30 s at 2500 r/min. Following centrifugation at 2500 r/min for 10 min at 4 °C, the plasmid DNA chitosan nanoparticles were collected from the precipitate, and were dissolved in phosphate buffered saline (PBS, pH 7.4). The final nanoparticles were named the pcDNA3.1 (+)-*cagW*-CS-NPs.

### Characterization of the pcDNA3.1 (+)-*cagW*-CS-NPs

Scanning electron microscope (SEM) (Tescan, Vega3 series, USA) was applied to evaluate pcDNA3.1 (+)-*cagW*-CS-NPs in terms of morphological and surface characteristics. A Zeta Sizer 2000 (Malvern, UK) determined the zeta potentials and dimensional size of the pcDNA3.1 (+)-*cagW*-CS-NPs. Samples were dissolved in water, and subsequently, calculations were performed at a scattering angle of 90° in 25 °C. Intensity of light scattered from particles characterized the diameter through auto-correlation function, assuming a spherical form of the particles.

### Stability of the pcDNA3.1 (+)-*cagW*-CS-NPs

The stability assessment of the pcDNA3.1 (+)-*cagW*-CS-NPs was based on gel retardation method. Briefly, 1 μg of pure plasmid DNA and the pcDNA3.1 (+)-*cagW*-CS-NPs containing 1 μg of plasmid DNA were incubated with DNase I (1.0 U/ml) at 37 °C for 30 min. To stop the reaction, 1 µl EDTA was added to the solution, and was incubated at 65 °C for 5 min. Then, 16 μl of chitosanase (0.2 U/ml) and 4.0 μl of lysozyme (0.2 U/ml) were mixed in a 37 °C water bath for 1 h. pcDNA3.1 (+), and the mixture was regarded as negative control. The integrity of plasmid DNA was analyzed using agarose gel electrophoresis.

### Transfer into HDF cell

When the confluency of HDF cells reached 80–85%, HDF were seeded into 6-well plates for transfections. Lipofectamine 2000 reagent (Invitrogen, USA) and recombinant pcDNA3.1 (+)-*cagW*-CS-NPs, pcDNA3.1 (+)-*cagW* and pcDNA3.1 (+) were mixed by Opti-MEM separately. The mixture of diluted vector and lipofectamine in 1:1 ratio was incubated for 5 min at room temperature. The lipid-DNA complex was then added to the HDF cells in a serum-free DMEM for 6 h. For selection, the serum-free DMEM was then replaced with a fresh medium containing 50 g/ml- Neomycin. The HDF cells transfected with pcDNA3.1 (+) were regarded as control of transfection.

### Western blotting

After 36 h post-transfection, the samples derived from recombinant pcDNA3.1 (+)-*cagW*-CS-NPs, pcDNA3.1 (+)-*cagW* and control pcDNA3.1 (+) were lysed in RIPA buffer. Extracted proteins were separately electrophoresed on 12% SDS-PAGE gels, and were transferred to nitrocellulose (GE Amersham Biosciences, Piscataway, NJ, USA). Rabbit anti-human *cagW* antibody at a 1:700 dilution [26] and anti-GAPDH antibody (ab 469) at a 1:700 dilution were incubated at 4 °C overnight. It is followed by adding goat anti-mouse Abs conjugated with HRP (Santa Cruz Biotechnology) to bind the first Abs. Finally, target proteins were detected by ECL reagents.

### Quantitative PCR

Total RNA from recombinant pcDNA3.1 (+)-*cagW*-CS-NPs and pcDNA3.1 (+)-*cagW* were extracted by the RNA extraction kit (Qiagen, USA). For each sample, RNA concentration was determined by Thermo Scientific™ NanoDrop 2000 at 260/280 nm RNA concentration. The complementary DNA synthesis was performed using the SuperScript™ First-Strand Synthesis System (Invitrogen, USA) in a reaction volume of 20 μl according to the manufacturer’s instructions. The best primer concentrations were identified by performing a series of experiments with varying primer combinations (Table 1). The specific primers were used to quantify *cagW* mRNA by RT-PCR kit (Takara, Japan) on a Rotor gene 6000 (Corbett Research, Sydney, AU). Gene expression was calculated through the 2^−ΔΔ*CT*^ method, and was normalized to GAPDH levels regarded as the internal control.

**Table 1:** Sequences and optimized concentration of primers used in this study

Primers	Primer sequence (5′ to 3′)	Primer concentrations (nM)		Ta (**°**C)	Product size (bp)
		Forward	Reverse		
GAPDH-F	TCCXGTAGACAAAATGGTGAAGG	900	900	61	261
GAPDH-R	ATGTTAGTGGGGTCTCGCTCCTG			61	
CagW-F	CACACCATTAGCCACAAGTTTAGC	300	600	61	223
*CagW*-R	ATGCGTTCCAACCAAAATTACAG			61	

### Immunofluorescence assay

After 36 h post-transfection, immunofluorescence assays (IFA) were used to detect the expressions of *cagW* proteins. Briefly, the cells fixed with 4% paraformaldehyde for 24 h were incubated with the primary mouse polyclonal antibody (1:200 dilutions in 0.1 M PBS) against *cagW* for 1 h. Following 3 times wash with PBS, the cells were incubated with 3% BSA for 30 min for blocking. Fluorescein isothiocyanate (FITC)-labeled goat anti-mouse IgG (Origene, Rockville, MD, USA) (1:200 dilution in 0.1 M PBS) was incubated for 1 h at room temperature. Cells were washed with PBS, and Hoechst 33,342 dye (1 μg/ml; Sigma) was added for 5 min in the dark. The green and blue fluorescence signals were observed under inverted fluorescence microscope (Leica, Germany). The primary antibody was replaced with 3% BSA in PBS as negative controls.

### Mice models

Animal protocols were based on the guidelines of the Declaration of Helsinki (1964), and animal studies were approved by Islamic Azad University of Shahrekord, Shahrekord, Iran. BALB/c mice (6–8 weeks-old) purchased from Animal House of Pasteur Institution were used, and they were kept in pathogen free condition. All mice were divided into four groups, and were treated on days 0, 7, 14, 30 and 45. Mice were injected with intramuscular injection of 100 µg/mouse of pcDNA3.1 (+)-*cagW*-CS-NPs and pcDNA3.1 (+)-*cagW* vaccines. Control mice were treated by 100 µg empty vector (pcDNA3.1 (+)) and 100 ml PBS.

### IgG and IgM antibody in serum

Serum samples were collected from all groups before vaccination 0, 7, 14, 30 and 45 days after injection (0.33 µg/µl × 3 = (100 µg/µl)). The level of IgG and IgM was evaluated by ELISA method according to the manufacturer instructions. First, 10 µl from each sample was added to the specific coated wells followed by washing and incubating goat anti-mouse secondary antibody conjugated with horse-radish peroxidase) HRP). After that, plates were washed and optical density was detected at 460–630 nm.

### Detection of cellular immune response

The cellular immune response was performed with regard to the proliferation of lymphocytes, the number of CD4^+^ and CD8^+^ T subset lymphocytes and the levels of IFN-γ, IL-2, IL-12 and IL-4 cytokines. The peripheral blood mononuclear cells (PBMCs) from the spleen or peripheral blood were extracted, and then lymphocytes were isolated by Ficoll density gradient centrifugation, and were separated from plasma. The MTT (3-[4,5-dimethylthiazol-2-yl]-2,5-diphenyl tetrazolium bromide) assay was applied to determine the proliferation of the lymphocytes in triplicates. The population of CD4^+^ and CD8^+^ T lymphocyte cells was sorted by flow cytometry. Lymphocytes were incubated with FITC-conjugated anti-CD4^+^ T cell antibody and phycoerythrin (PE)-conjugated anti-CD8^+^ T cell antibody (1:1000 dilution) (Sungene, China) at 4 °C for 1 h. Then, the cells were washed 3 times with cold PBS, and were assessed by flow cytometry. In serum samples and spleen suspension, the IFN-γ, IL-2, IL-12, and IL-4 levels were evaluated using ELISA kits (ABCAM) based on standard curves.

### Challenge experiments

To investigate the protective efficacy of different DNA vaccines for *H. pylori*, all groups were challenged with 1 × 10^9^ CFU *H. pylori* by p.o. after giving 200 μl of 0.2% sodium bicarbonate solution. The mortality of the challenged mice was monitored for the subsequent 30 days. The bacterial burdens in the liver and gastric were detected at day 7 post infection as previously described [27]. Briefly, the mice were euthanized at day 7 post infection gastric tissue was cut. The liver was aseptically removed and was macerated by passage through a 3-ml syringe. Gastric tissue and liver were incubated on a brain heart infusion (BHI, BD Difco, NJ, USA) agar medium containing 5% fetal bovine serum (FBS, GE Healthcare, NJ, USA.) at 37 °C for 48 h, and bacterial viable counts were determined. Infection was measured as either mortality or the presence of ≥ 500 CFU of *H. pylori* per g of liver and per g of gastric tissue during necropsy.

### Histological analysis

After procedures for the necropsy at day 0 post infection, the livers of all groups of immunized mice were aseptically harvested and fixed in 10% formalin. The paraffin-embedded tissue sections were prepared on a rotary microtome, and were stained with hematoxylin–eosin by using standard techniques [28]. All sections were examined by light microscopy. Triplicates were completed for each control and sample.

### Statistical analysis

GraphPad Prism 5.0 was applied to analyze data and statistical tests. Multiple comparisons between three groups were made by Tukey post hoc. Unpaired *t* Test was used to compare the survival rates between immunized mice and the control group. Means were compared by using a one-way analysis of variance (ANOVA), followed by a Tukey–Kramer post hoc test using a 95% confidence interval. Differences were considered significant at *p* < 0.05.

## Results

### Construction and identification

**Fig. 1:**
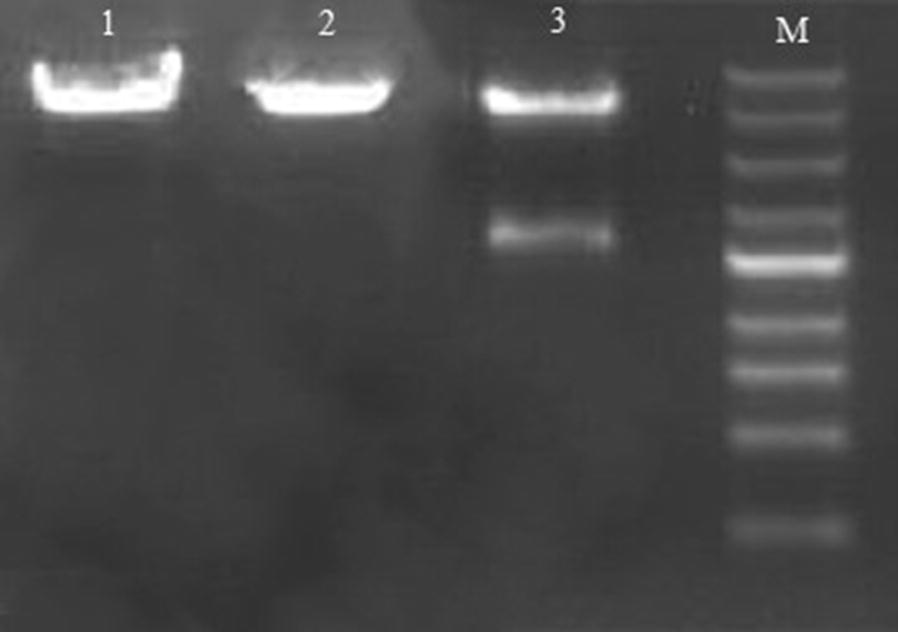
The pcDNA3.1 (+)-*cagW*, pcDNA3.1 (+) and the digestion product were separated by electrophoresis. Lanes 1 and 2 depict the 7039 and 5428 bp fragments of pcDNA3.1 (+)-*cagW* and pcDNA3.1 (+), respectively, Lane 3 is BamHI/EcoRV restriction digest on pcDNA3.1 (+)-*cagW* plasmid, and lane M is 1 Kb DNA ladder (Thermo Fisher Cat No. 10816-015)

As noticed in Fig. 1, *cagW* gene was successfully inserted into the pcDNA3.1 (+) vector, and it led to the DNA vaccine plasmid pcDNA3.1 (+)-*cagW*, which was exposed to digestion with BamHI and EcoRV. The expression of 5428 and 1611 bp was related to pcDNA3.1 (+) and *cagW* amplicon, respectively.

### Chitosan enhanced stability of the pcDNA3.1 (+)-*cagW*-CS-NPs

**Fig. 2:**
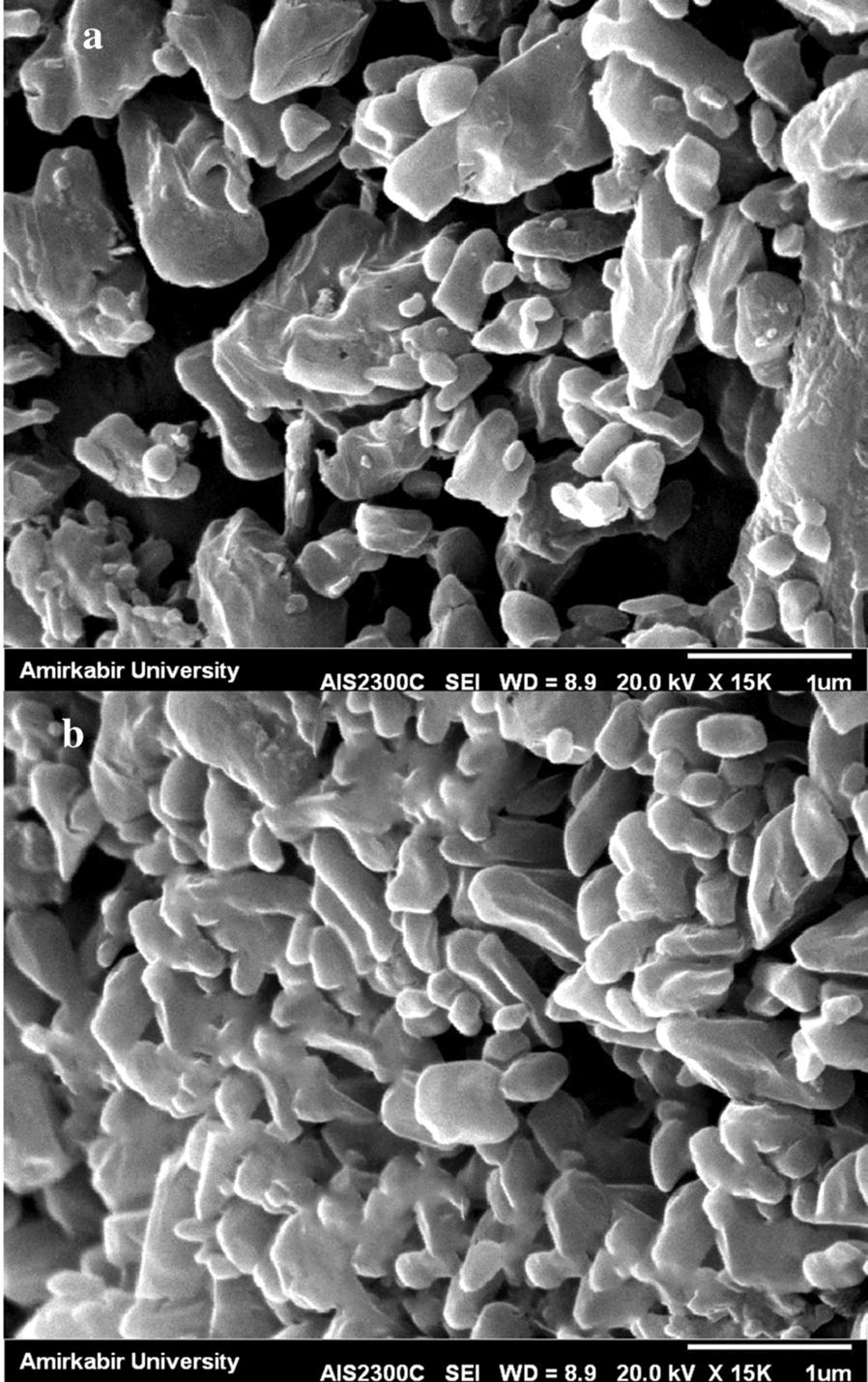
Scanning electron microscope micrograph: (A) CS-NPs (B) the pcDNA3.1 (+)-*cagW*-CS-NPs

The DNA vaccine that was encapsulated in chitosan nanoparticles was prepared in accordance with complex coacervation in order to determine the stability of pcDNA3.1 (+)-*cagW*-CS-NPs. SEM observation showed the spherical poly disperse nature of pcDNA3.1 (+)-*cagW*-CS-NPs. Figure 2 illustrates that the morphology of pcDNA3.1 (+)-*cagW*-CS-NPs is round with a flat surface and suitable dispersion without any adhesion. The average thickness was 68.84 nm, the particle-size (Z-Average) was 117.7 nm and the zeta potential was +5.64 mV, and the pure plasmid DNA was degraded for 30 min when it was incubated with DNase I. The impact of protection to plasmid DNA from DNase I degradation was determined by DNase I and chitosanase. The results reveal that chitosan-DNA complex presents a notable protection of the plasmid.

### Plasmids induced inhibition of proliferation

**Fig. 3:**
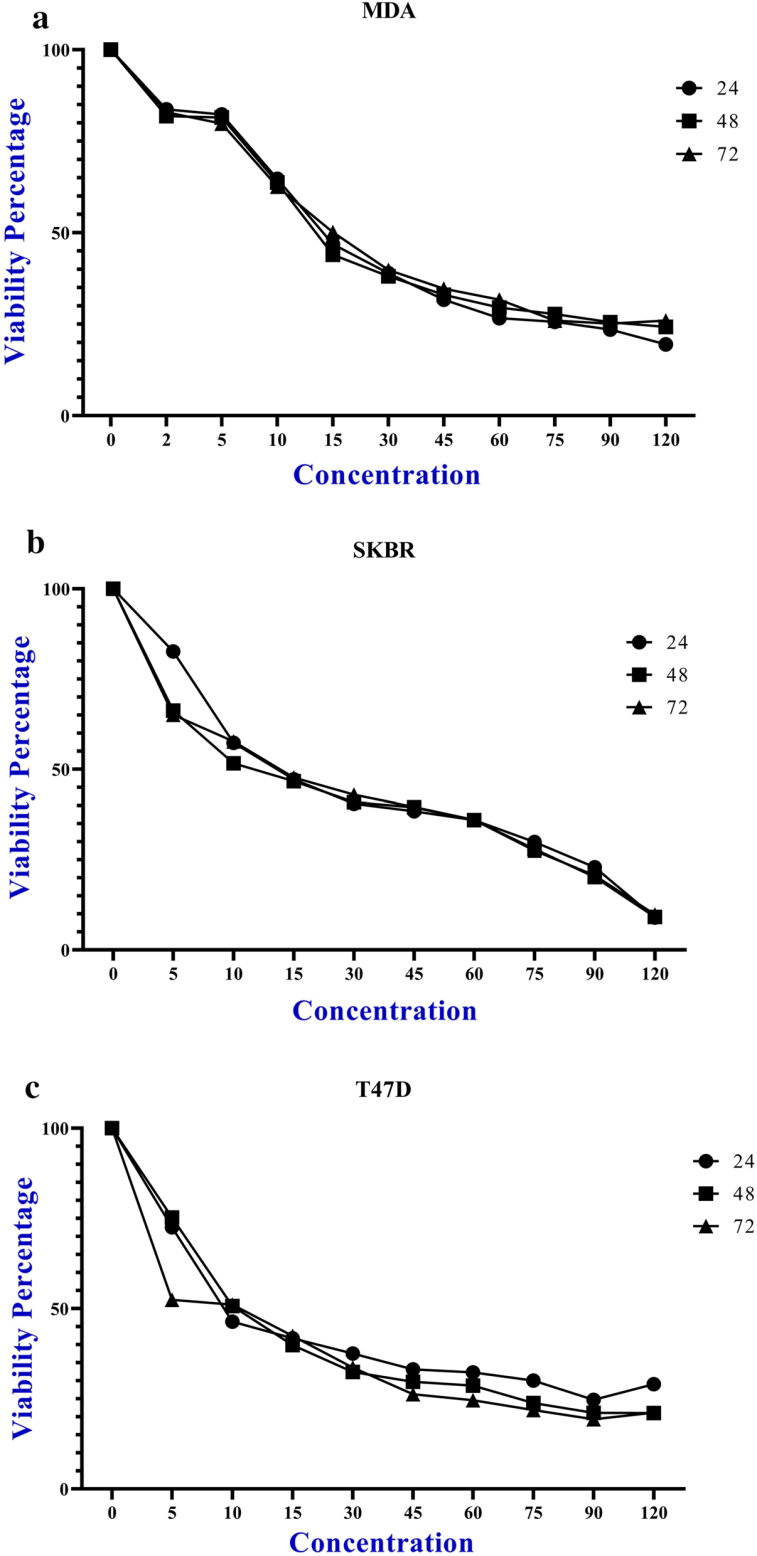
Plasmids decreased growth and proliferation of HDF cell line in concentration-dependent manner in all the time of incubation (µg/ml): **a** pcDNA3.1 (+), **b** pcDNA3.1 (+)-cagW and **c** pcDNA3.1 (+)-cagW-CS-NPs

Cytotoxicity effect of plasmids i.e. pcDNA3.1 (+), pcDNA3.1 (+)-*cagW* and pcDNA3.1 (+)-cagW-CS-NPs was evaluated on HDF cell line. HDF was treated with different concentration plasmids for incubation time of 24, 48 and 72 h. MTT was used to measure cell viability (Fig. 3).

### Expression of *cagW* in cell line

**Fig. 4:**
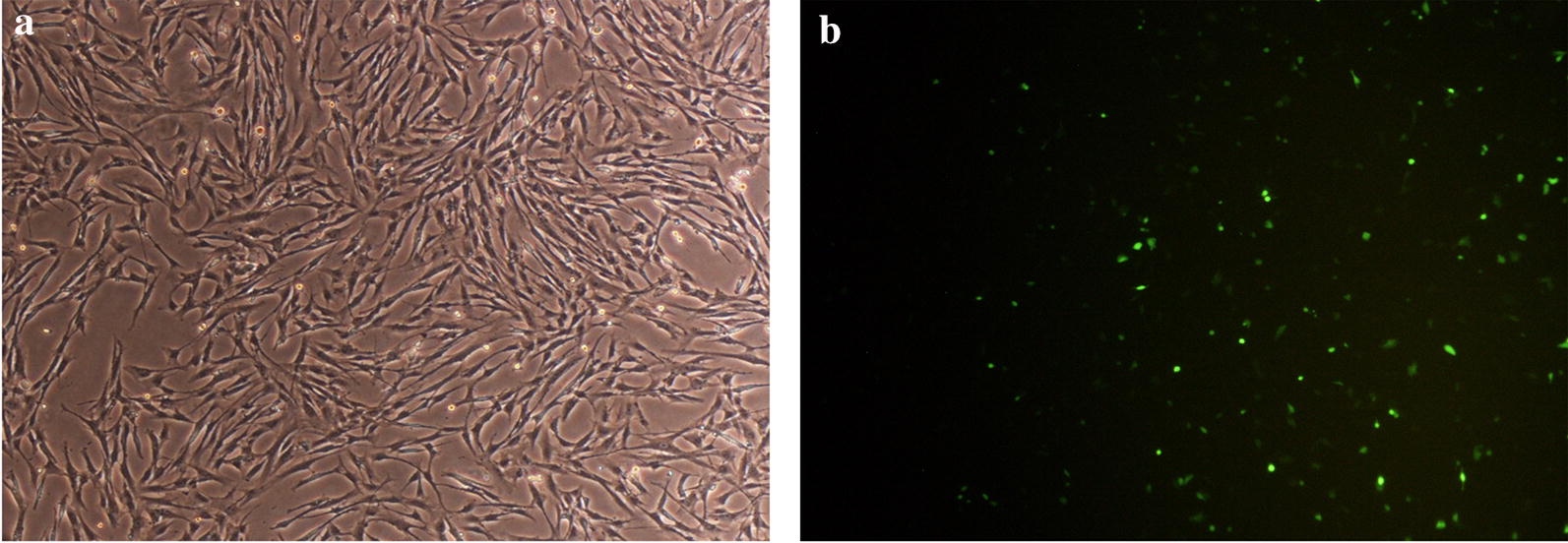
Transient expression of with pcDNA3.1 (+)-cagW-CS-NPs for HDF cells transfected with pcDNA3.1 (+)-cagW-CS-NPs: **a** Cell controls. **b** Cells were transfected with vector pcDNA3.1 (+)-cagW-CS-NPs

**Fig. 5:**
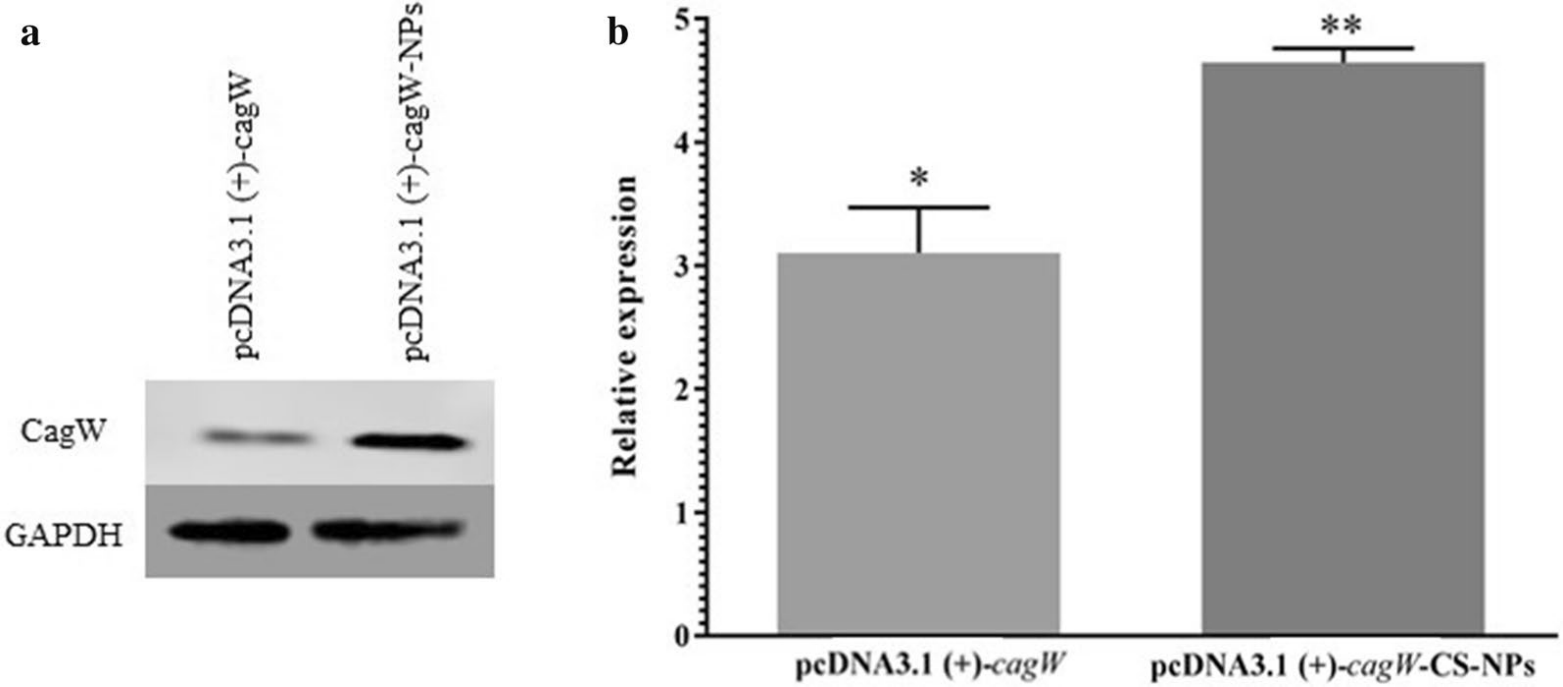
Relative expression of *cagW* gene in pcDNA3.1 (+)-*cagW*-CS-NPs and pcDNA3.1 (+)-*cagW* in HDF cell line by the DDCT method. *significant at the 0.05 level; **significant at the 0.001 level; ***significant at the 0.0001 level. Error bars show the minimum and maximum variables

IFA assay was used to confirm the expression of *cagW* protein in transfected *HDF* cells. The green fluorescence was noticed for *HDF* cells that were transfected with pcDNA3.1 (+)-*cagW*-CS-NPs, however, cells that were transfected with empty vector and blank control did not have any observable fluorescence. In addition, as reported in the previous investigations, IFA was employed to detect the expression of *cagW* by mouse polyclonal antibody. The findings revealed that the green fluorescence was also recognized by inverted fluorescence microscopy for HDF cells that were transfected (Fig. 4). Furthermore, the antigen expression was investigated by Western blot (Fig. 5a), showing that the transfection of pcDNA3.1 (+)-*cagW* increased the amount of cagW protein in comparison to control. Correspondingly, the results revealed that transfection with pcDNA3.1 (+)-*cagW*-CS-NPs remarkably increased the mRNA level of *cagW* in comparison to pcDNA3.1 (+)-*cagW* (Fig. 5b).

### Expression level of *cagW* gene in tissue

**Fig. 6:**
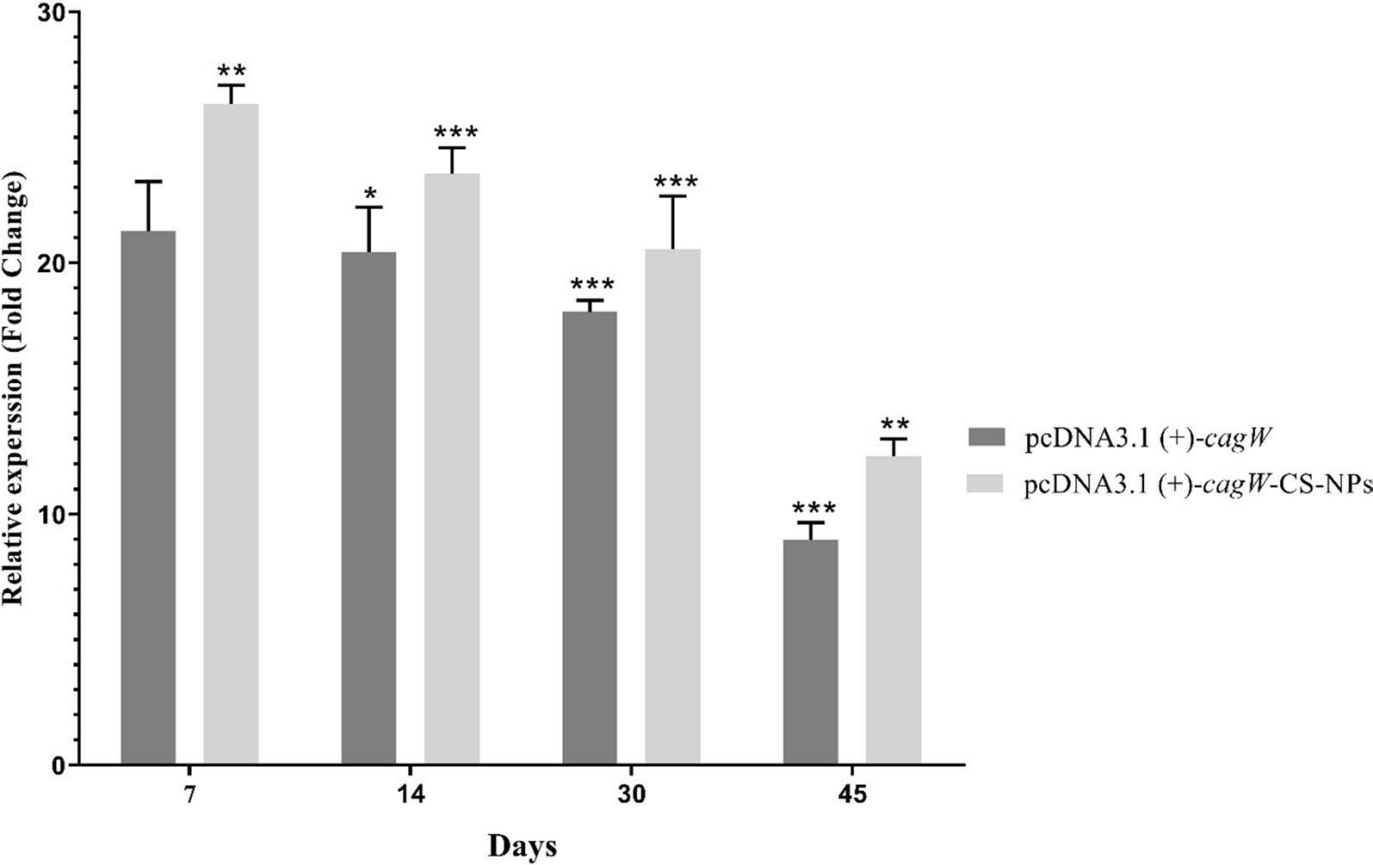
Expression levels of *cagW* gene DNA vaccine in different times (7, 14, 30 and 45). qRT-PCR was employed inorder to quantify the expression levels of *cagW* gene DNA vaccine on different days. *significant at the 0.05 level; **significant at the 0.001 level; ***significant at the 0.0001 level. Error bars show the minimum and maximum variables

Using qRT-PCR, the stability rate of *cagW* gene DNA vaccine was assessed in different times after the last injection (0, 7, 14, 30 and 45). In comparison to average expression in different times, *cagW* rate on day 7 was upregulated in pcDNA3.1 (+)-*cagW*-CS-NPs and pcDNA3.1 (+)-*cagW*, whereas 14 and 30 times was downregulated then 7 time (Fig. 6). *CagW* rate in 45 time showed lower expression levels in pcDNA3.1 (+)-*cagW*-CS-NPs and pcDNA3.1 (+)-*cagW*.

### Production of antibody by DNA vaccines

**Fig. 7:**
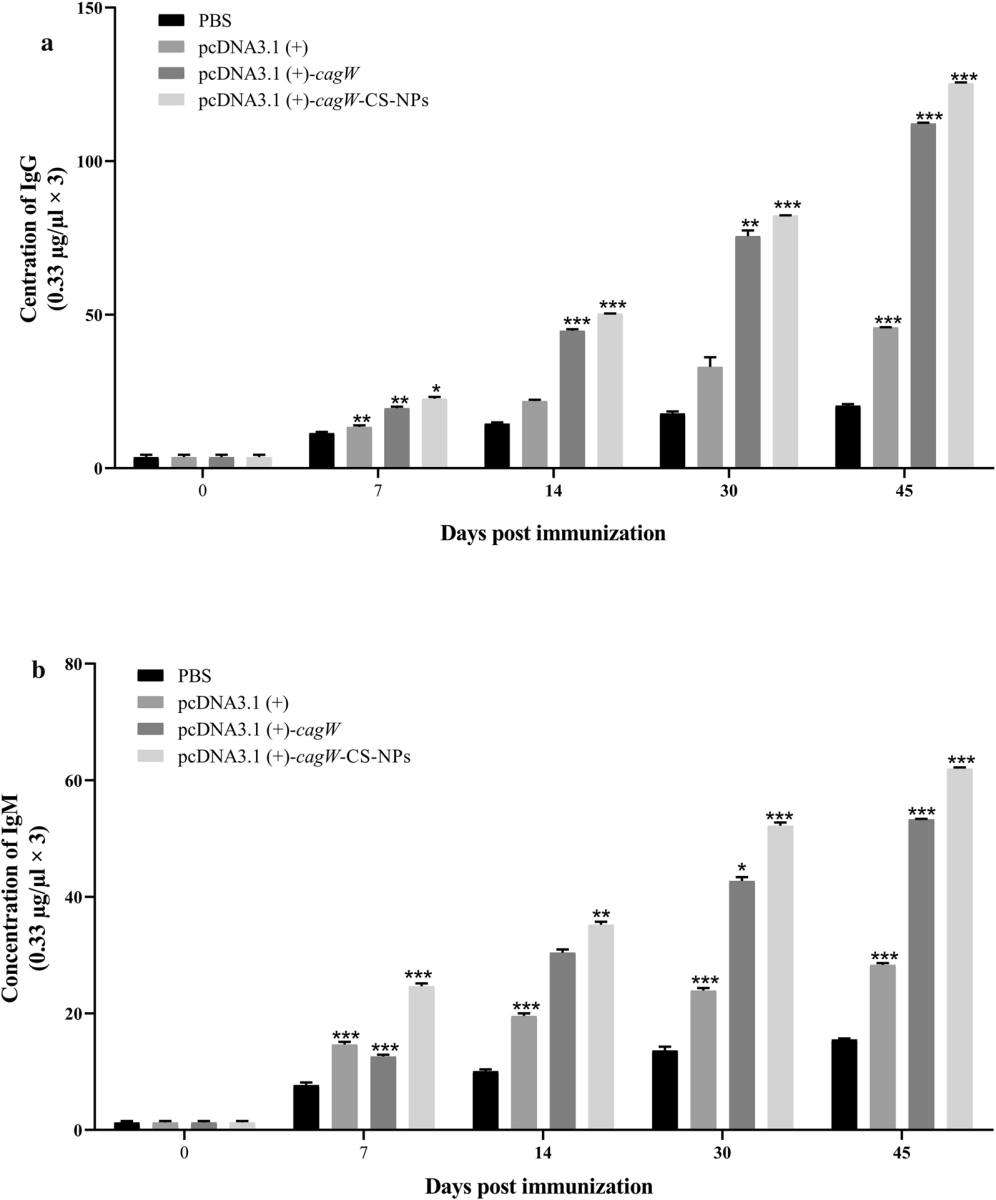
Humoral immune responses induced by plasmids. Mice were immunized with pcDNA3.1 (+)-*cagW*-CS-NPs and pcDNA3.1 (+)-*cagW*, and were boosted once with a 3-week interval with PBS used as a control. Serum samples were collected from mice’s tail veins at indicated time points, and the titers of the IgG (**a**) and IgM (**b**) antibodies were detected by indirect ELISA assay. Data showed mean ± SD of OD at 490 nm. *p < 0.05 and **p < 0.01 (determined by one-way ANOVA followed by a Tukey–Kramer post hoc test)

pcDNA3.1 (+)-*cagW*-CS-NPs and pcDNA3.1 (+)-*cagW* were prepared. Each of them surrounded the *cagW* gene in order to test if the immunogenicity of a pcDNA3.1 (+)-*cagW*-CS-NPs vaccine targeting *H. pylori* was higher than the one that was elicited by pcDNA3.1 (+)-*cagW*-CS-NPs and pcDNA3.1 (+)-*cagW* vaccines. Mice were injected in in their muscles with either PBS control, pcDNA3.1 (+), pcDNA3.1 (+)-*cagW* plasmid or pcDNA3.1 (+)-*cagW* chitosan-DNA nanoparticle. The titers of IgG and IgM serum antibodies were measured by ELISA at regular intervals. As illustrated in Fig. 7a, the production of IgG serum antibody titers in sera on day 45 was induced by vaccination with the pcDNA3.1 (+)-*cagW*-CS-NPs and pcDNA3.1 (+)-*cagW*. Antibody could be detected when the mice were vaccinated with pcDNA3.1 (+)-*cagW*-CS-NPs and pcDNA3.1 (+)-*cagW* since day 45 showed a higher titer on the indicated time points (p < 0.05); whilst in PBS groups, no or low antibody titers in sera could be detected. The results revealed that pcDNA3.1 (+)-*cagW*-CS-NPs vaccine induced higher hormone immune response than pcDNA3.1 (+)-*cagW* and pcDNA3.1 (+), and the serum antibody titers IgG was higher than IgM subtype (Fig. 7b), which suggested that these types of plasmids were responsible for the induction of Th1 type immune response.

### The serum antibody titers

#### Enhancement of cellular immune response by DNA vaccines

**Fig. 8:**
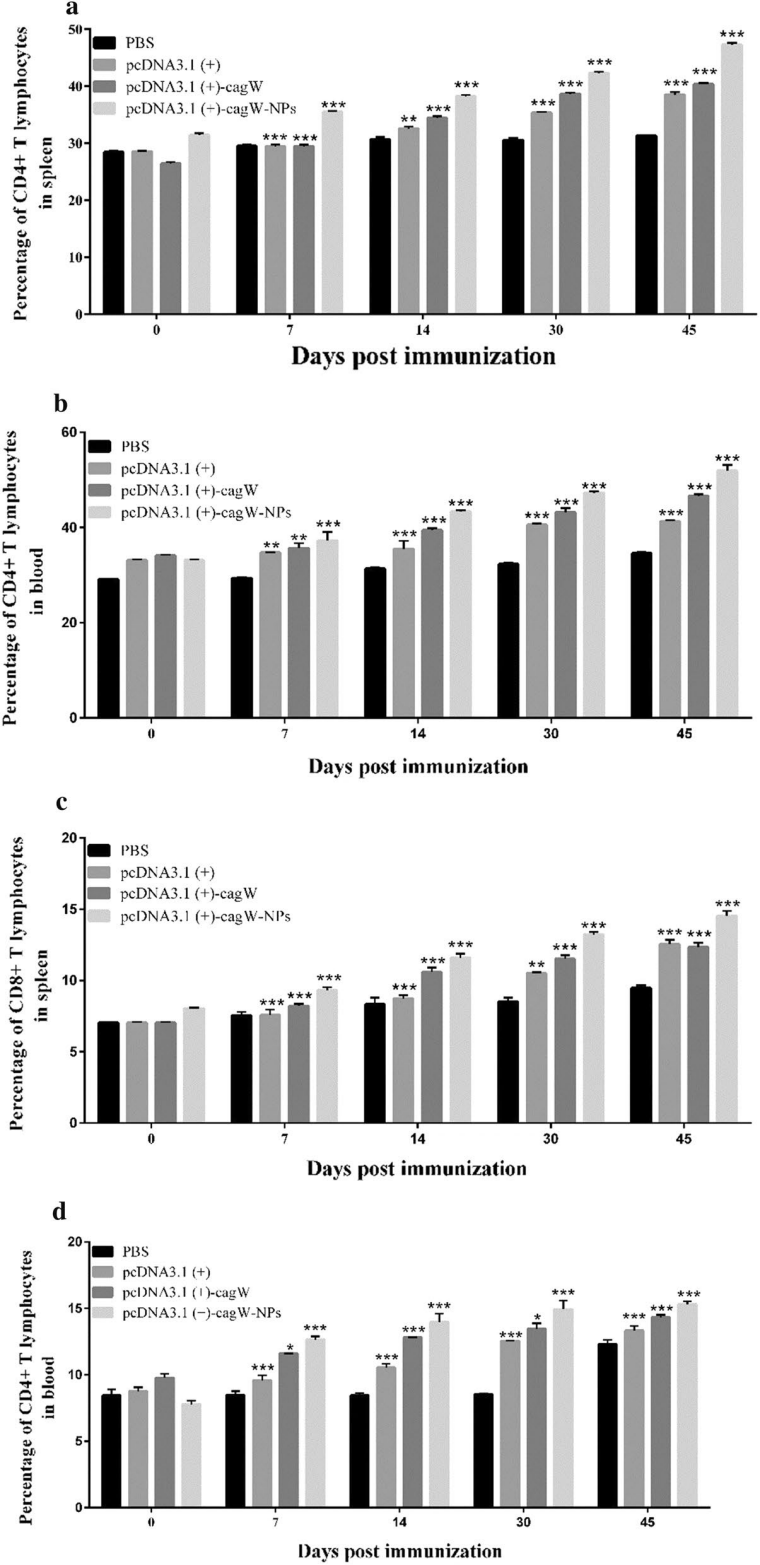
Spread of T lymphocytes and changes in CD4^+^ and CD8^+^ T lymphocytes in the spleens and peripheral blood. The CD4^+^ T Lymphocytes were separated from the spleen (**a**), CD4^+^ T Lymphocytes were separated from the blood (**b**), CD8^+^ T Lymphocytes were separated from the spleen (**c**) and CD4^+^ T Lymphocytes were separated from the blood (**d**). The CD4^+^ and CD8^+^ T Lymphocytes were separated from the spleen and peripheral blood at specified time points, and were sorted by flow cytometry with PBS as a control. Data showed mean ± SD. *p < 0.05, **p < 0.01, and ***p < 0.001 (determined by one-way ANOVA followed by a Tukey–Kramer post hoc test)

The changes of CD4^+^ and CD8^+^ T lymphocytes and the levels of IFN-γ, IL-2, IL-4 and IL-12 induced by the plasmids were studied in order to assess the cellular immune response. As depicted in Fig. 8, the spread of lymphocytes and the level of IFN-γ, IL-2, IL-4 and IL-12 of mice that were immunized with pcDNA3.1 (+)-*cagW*-CS-NPs increased compared to other groups. The results of MTT assay revealed that the values from the groups of pcDNA3.1 (+)-*cagW*-CS-NPs and pcDNA3.1 (+)-*cagW* were increased between 7 and 45 days, while these effects were not noticed in the PBS groups. Immunization with pcDNA3.1 (+)-*cagW*–NPs created more lymphocytes in the spleen in comparison to other groups (p < 0.001). The spread of lymphocytes showed a similar tendency between 7 and 45 days in peripheral blood (p < 0.001). In order to determine the changes of CD4 + and CD8 + T lymphocytes in the spleen and blood, flow cytometry was applied. As seen in Fig. 8, the percentages of CD4 + and CD8 + T lymphocytes in spleens increased after the mice were immunized with plasmids pcDNA3.1 (+) and pcDNA3.1 (+)-*cagW* plasmid (p < 0.001) in comparison to the control groups (immunization with pcDNA3.1 (+)-*cagW*-CS-NPs (p < 0.001) between 7 and 45 days), and the group of pcDNA3.1 (+)-*cagW*-CS-NPs showed a higher level than other groups. Moreover, changes in CD4 + and CD8 + T lymphocytes were increased in the blood between 30 and 45 days.

**Fig. 9:**
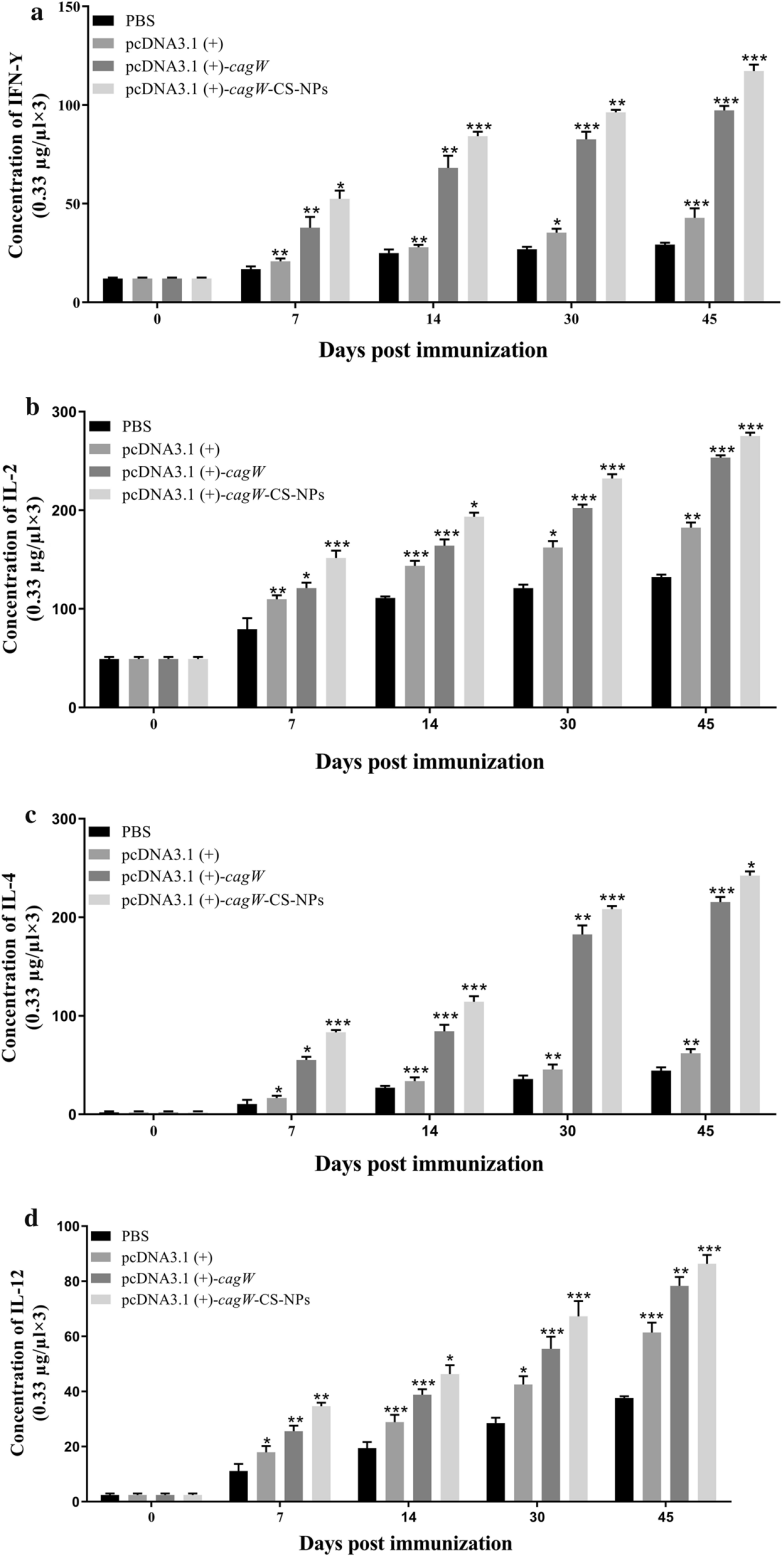
Levels of IFN-γ, IL-2, IL-4 and IL-12 from suspension of spleen lymphocytes in immunized mice. Levels of IFN-γ (**a**), IL-2 (**b**), IL-4 (**c**) and IL-12 (**d**) from the suspension of spleen lymphocytes were determined by ELISA assays, and PBS was used as a control. Data showed mean ± SD. *p < 0.05, **p < 0.01, and ***p < 0.001 (determined by one-way ANOVA followed by a Tukey–Kramer post hoc test)

The levels of IFN-γ, IL-2, IL-12, and IL-4 were assessed by using ELISA. The IFN-γ, IL-2, IL-12, and IL-4 levels were enhanced by immunization with pcDNA3.1 (+)-*cagW*-CS-NPs, pcDNA3.1 (+)-*cagW* pcDNA3.1 (+) and PBS alone in the suspension of the spleen lymphocytes. In addition, the results revealed that the vaccination of encapsulated pcDNA3.1 (+)-*cagW* in chitosan nanoparticles raised the levels of IFN-γ, IL-2, IL-4 and IL-12 (Fig. 9). Upon immunization of the mice with pcDNA3.1 (+)-*cagW*-CS-NPs, higher levels of IFN-γ, IL-2, IL-4 and IL-12 were observed between 7 and 45 days compared to other groups (p < 0.001). The levels of IFN-γ, IL-2, IL-4 and IL-12 revealed significant changes in the suspension of the spleen lymphocytes of the mice that were immunized with PBS. These results clearly revealed that pcDNA3.1 (+)-*cagW*-CS-NPs induced effective immune responses, and that the structure of the pcDNA3.1 (+)-*cagW*-CS-NPs could serve as a molecular assistant in relation to DNA immunogens in order to ameliorate the cellular immune responses.

#### Protection mice from *H. pylori* challenge

**Fig. 10:**
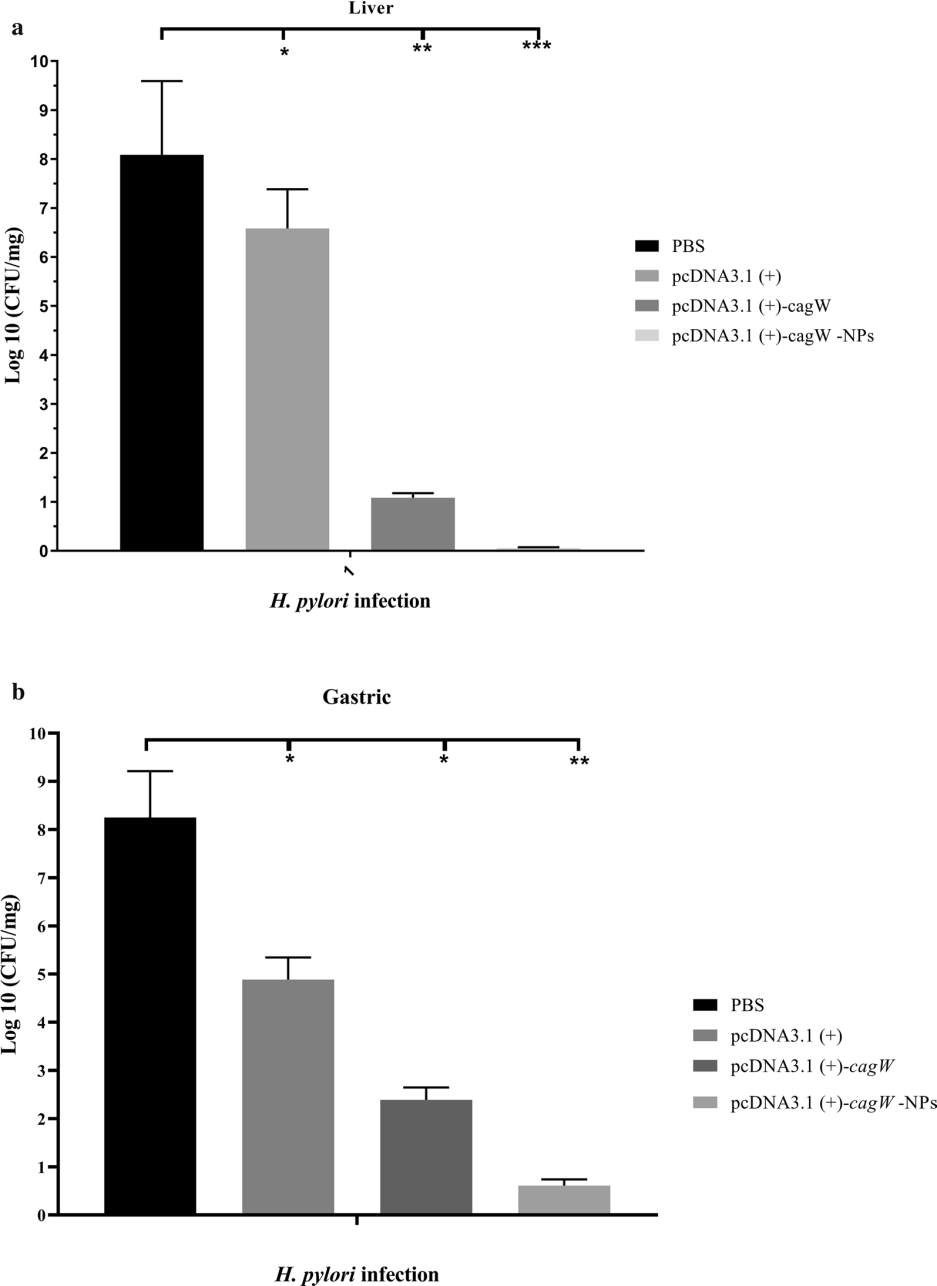
CFUs rate of mice challenged intraperitoneally with *H. pylori*. (**a**) liver and (**b**) gastric. *significant at the 0.05 level; **significant at the 0.001 level; ***significant at the 0.0001 level. Error bars show the minimum and maximum variables

The CFU of viable bacteria in the liver or gastric was examined after intraperitoneal challenge with *H. pylori* in order to recognize the ability of chitosan-DNA vaccine-raised immunity to remove *H. pylori* infection. The mice were challenged with 1 × 10^9^ CFU *H. pylori*, a lot of bacteria from the liver and gastric were recovered in the mice treated with PBS and CS-NP. In contrast, all the mice that were vaccinated with pcDNA3.1 (+)-*cagW*-CS-NPs had few or no bacteria in the liver (p < 0.001) (Fig. 10a) and gastric (p < 0.05 or p < 0.001) at 30 days after infection (Fig. 10b). In addition, DNA vaccination presented a significant clearance of bacteria. In comparison to the bivalent DNA vaccine group, the PBS and pcDNA3.1 (+) of immunized mice died on day 3 or day 7, but almost half of the mice that were vaccinated with pcDNA3.1 (+)-*cagW*-CS-NPs and pcDNA3.1 (+)-*cagW* survived the *H. pylori* challenge on day 30 (p < 0.001). Apart from that, 100% of mice that were immunized with pcDNA3.1 (+)-*cagW*-CS-NPs and pcDNA3.1 (+)-*cagW* had no disease symptoms at any time and survived against *H. pylori* challenge on day 45 (p < 0.001). From histological view, the livers of the mice in the control group showed a necrotic center which contained leukocytes, hepatocytes and cellular debris. The interesting point was that the immunized mice removed the invading bacteria from their livers during *H. pylori* challenge, and their liver tissues had no obvious pathological changes in comparison to control group. Moreover, pcDNA3.1 (+)-*cagW* vaccination showed fewer pathological changes in comparison to the bivalent DNA vaccine group. In brief, these results reveal that pcDNA3.1 (+)-*cagW*-CS-NPs and pcDNA3.1 (+)-*cagW* vaccines protect the mice from *H. pylori* challenge effectively.

## Discussion

*Helicobacter pylori*, a causative agent of gastritis, duodenal ulcers, gastric ulcer and cancer [29], is an opportunistic and infectious bacterium not only in humans, but also in animals. The infection rates are over 50.8% in developing countries whereas they are more than 34.7% in developed countries [30]. Because of the worldwide dissemination of multiple antibiotic resistance strains [31], the treatment of infections brought about by this pathogen has become a remarkable clinical challenge. In addition, since the development of new antimicrobial reagents has also been challenging, a different approach might be a viable alternative [31, 32] by using the immunological characteristics of some antigens of *H. pylori*. The complex pathogenicity of *H. pylori* and the diverse functions of their virulence factors are major obstacles to the development of effective universal vaccines. In this research, pcDNA3.1 (+)-*cagW*-CS-NPs was successfully prepared using an coacervation method. It had strong humoral and cellular immune responses and protective effects against *H. pylori* infections. Chitosan-DNA vaccines which target different components of bacteria can synergistically act to provide a host with the range of protective immunity against *H. pylori*. Chitosan nanoparticles (CNPs) can be administrated through oral, nasal, pulmonary, and ocular routes which are non-invasive [33]. In addition, they have been proposed as non-viral vectors in gene therapy and have proved their adjuvant impacts in vaccines [34]. By combining CNPs with monoclonal antibody with reduction of drug side effects in some specific cancer patients, they are also used for chemotherapy drug delivery [35]. CNPs may be safe and effective adjuvant candidates appropriate for therapeutic vaccines. Experiments have revealed that CNPs are potentially strong to increase cellular and humoral immune responses, and elicit a balanced Th1/Th2 response. Wen et al. studied the promoted immune response to ovalbumin (OVA) in mice by CNP and its toxicity [36]. In their study, the mice were immunized subcutaneously with OVA alone, or with OVA that was dissolved in saline containing Quil A, chitosan, or CNP, and CNP did not bring about any side effects or cell mortality. They noticed that the serum OVA-specific IgG, IgG1, IgG2a, and IgG2b antibody titers and Con A-, LPS-, and OVA-induced splenocyte proliferation were remarkably increased by CNP compared to OVA and chitosan groups, and CNP remarkably increased the killing activities of NK cells activity [37]. It also promoted the production of Th1 (IL-2 and IFN-*γ*) and Th2 (IL-10) cytokines, and upregulated the mRNA expression of IL-2, IFN-*γ*, and IL-10 cytokines in splenocytes from the immunized mice in comparison to OVA and chitosan groups [37, 38]. Wu et al. proved that humoral and cellular immunities were remarkably increased in immunized mice that survived by resisting the infection of *E. coli*, whereas the control mice showed clear symptoms and lesions of infection [37]. Their results proved that inoculation with CpG-CNP remarkably increased the content of IgG, IgM, and IgA in the immunized mice’s sera. White blood cells, lymphocytes and elevated levels of IL-2, IL-4, and IL-6 were also noticed in the mice in CpG-CNP group, indicating that CpG-CNP can serve as an effective adjuvant in order to improve the immune protection and resistance of porcine against infectious diseases [39]. In addition, the particle size of chitosan has a vital role in regulating the immune response. In another report, small-sized chitin or chitosan particles activated alveolar macrophages to express cytokines like IL-12, tumor necrosis factor-*α* (TNF*α*), and IL-18, which led to INF-*γ* production [38]; however, in our study, small-sized pcDNA3.1 (+)-*cagW*-CS-NPs induced more potent lymphocyte proliferation and cytokine production in immunized mice than control group, which suggests that chitosan has complex and size-dependent effects on immune responses. Hence, it can be concluded that nanoform may function as a potent adjuvant in relation to DNA immunogens in order to enhance the immune responses, which may provide a protection from *H. pylori* challenges.

It has been proved that biodegradable polymers could be used as delivery vectors for antigen genes and materialize sustained release [40]. In this research, the *cagW* gene DNA vaccine, encapsulated in high-quality chitosan nanoparticles, was prepared. Under optimal conditions, the pcDNA3.1 (+)-*cagW*-CS-NPs had a smooth surface, good dispersity, and no adherence or collapse phenomenon; moreover, the pcDNA3.1 (+)-*cagW*-CS-NPs protected the plasmid DNA from DNase I digestion, as illustrated by electrophoresis after the enzyme digestion. According to our findings, a recent research also found that chitosan nanoparticles were effective delivery vectors for DNA vaccines and materialize sustained release [41]. Moreover, the in vitro HDF cells that were transfected with the pcDNA3.1 (+)-*cagW*-CS-NPs showed that the expression of specific antigens was successfully detected, confirming the activation of *cagW* gene. These findings revealed that the procedure of nanoparticle production was safe, and the bioactivity of the plasmid DNA persisted after the nanoparticles were produced.

Analyses of IgG antibody responses from the pcDNA3.1 (+)-*cagW*-CS-NPs, pcDNA3.1 (+)-*cagW*-CS-NPs, and pcDNA3.1 (+) showed that intramuscular immunization with multivalent DNA vaccines induced stronger antibody responses than the control group, and immunization with pcDNA3.1 (+)-*cagW*-CS-NPs induced more specific antibody in immunized mice than other groups. Intramuscular immunization is very effective in eliciting humoral and cellular immune responses. Previous researches have proved that virulence genes enhance the immunity of the vaccine, and chitosan provides longer residence times by intramuscular infection [29, 42], allowing the vaccine to access the lymphoid tissue better and resulting in increased IgG production. Therefore, our study further confirmed the stimulation effect of humoral immune responses of *cagW* and chitosan in mice in comparison to the background signal stimulated by pcDNA3.1 (+) and PBS. Our previous research had shown that the Th1 type immune response was considered the major host response used to contain *H.pylori* infection. After the investigation of proliferation of lymphocyte and changes of CD4 + and CD8 + T lymphocytes, it was found that pcDNA3.1 (+)-*cagW*-CS-NPs induced more potent lymphocyte proliferation in immunized mice than pcDNA3.1 (+) and pcDNA3.1 (+)-*cagW* alone. At the same time, pcDNA3.1 (+)-*cagW*-CS-NPs remarkably increased more synthesis and release of IFN-γ, IL-2, IL-4 and IL-12 than other groups. Such interactions determine the protective mechanisms of the host that are essential against *H. pylori* infections and the development of particular antibacterial immunity. According to our findings, a recent study pointed out that chitosan increased dendritic cell maturation by inducing IFNs and enhanced cellular immunity [43].

## Conclusions

In this research, we successfully prepared pcDNA3.1 (+)-*cagW*-CS-NPs that induced remarkably higher mucosal and humoral immune responses and had protective effects against the challenge of *H. pylori* infections on the basis of a mouse model. In addition, the biodegradable polymer chitosan nanoparticles protected the plasmid DNA from degradation and increased the expression of encapsulated plasmid DNA. The findings reveal that the pcDNA3.1 (+)-*cagW*-CS-NPs encapsulated plasmid DNA is a safe and efficient drug release carrier system with a great potentiality for medical applications in the future. It is hoped that more comprehensive studies, by using controlled and targeted release of nanoparticles encapsulated DNA vaccine testing in large ruminants, will contribute to controlling *H. pylori* diseases.

## Data Availability

The datasets used and/or analyzed during the current study available from the corresponding author on reasonable request.
